# A Review of the Biology and Control of Whitefly, *Bemisia tabaci* (Hemiptera: Aleyrodidae), with Special Reference to Biological Control Using Entomopathogenic Fungi

**DOI:** 10.3390/insects11090619

**Published:** 2020-09-10

**Authors:** Ibrahim Sani, Siti Izera Ismail, Sumaiyah Abdullah, Johari Jalinas, Syari Jamian, Norsazilawati Saad

**Affiliations:** 1 Department of Plant Protection, Faculty of Agriculture, Universiti Putra Malaysia, Serdang 43400, Malaysia; sani.ibrahim@umyu.edu.ng (I.S.); izera@upm.edu.my (S.I.I.); sumaiyah@upm.edu.my (S.A.); 2 Department of Biology, Faculty of Natural and Applied Sciences, Umaru Musa Yar’adua University, P.M.B., Katsina 2218, Nigeria; 3 Laboratory of Climate-Smart Food Crop Production, Institute of Tropical Agriculture and Food Security (ITAFoS), Universiti Putra Malaysia, Serdang 43400, Malaysia; 4 Department of Biological Sciences and Biotechnology, Faculty Science & Technology, Universiti Kebangsaan Malaysia, Bangi 43600, Malaysia; johari_j@ukm.edu.my

**Keywords:** biological control, *Bemisia tabaci*, entomopathogenic fungi, host plant, whitefly

## Abstract

**Simple Summary:**

The whitefly, *Bemisia tabaci*, is considered one of the most destructive insect pests of vegetables and ornamental crops globally. Synthetic chemical pesticides are mainly used to control *B. tabaci*, however, their extensive usage has led to a series of detrimental concerns to human health and environmental contamination. It is therefore of significant interest to develop a safer and eco-friendly alternative for controlling *B. tabaci*. Here, we review the use of entomopathogenic fungi as a proven, biologically sustainable method to effectively control *B. tabaci.* The development of entomopathogenic fungi in an integrated pest management strategy against *B. tabaci* can reduce our reliance on chemical pesticides, and help us to secure food safety while preserving nature.

**Abstract:**

Whitefly, *Bemisia tabaci* (Gennadius) (Hemiptera: Aleyrodidae), consists of genetically diverse species known to cause significant destruction in several crops around the world. Nymphs and adults of *B. tabaci* cause damage to plants during feeding, and they can act as a virus vector, thus causing significant yield loss to crops in the tropical and subtropical regions. Chemical pesticides are widely used to control *B. tabaci* due to their immediate action, but this approach has several drawbacks including food safety issues, insecticide resistance, environmental pollution, and the effect on non-target organisms. A biological control agent using entomopathogenic fungi (EPF) has therefore been developed as an alternative against the conventional use of chemical pesticides in an integrated pest management (IPM) system to effectively control *B. tabaci*. It is apparent from this review that species of hyphomycetes fungi are the most common EPF used to effectively control *B*. *tabaci*, with the second instar being the most susceptible stage of infection. Therefore, this review article focuses specifically on the control of *B. tabaci* with special emphasis on the use of EPF as biological control agents and their integration in IPM.

## 1. Introduction

Of the 1556 species of whiteflies recorded in the world, *B. tabaci* remains one of the most economically important pests of vegetable and ornamental crops worldwide [1,2]. The insect feeds on several solanaceous and ornamental crops, including brinjal, chili, cotton, okra, potato, tomato, and tobacco [3,4]. The economic damage caused by *B. tabaci* ranges from mild to catastrophic with global annual loss reaching up to billions of USD in many crops [5,6,7]. *B. tabaci* adults are minute insects (usually 1 to 3 mm in length) that feed and oviposit in large quantities on the underside of leaves [8]. 

*Bemisia tabaci* may decrease the rate of photosynthesis in plants through the excretion of honeydew during feeding, besides being able to transmit a large number of plant pathogenic viruses including begomoviruses, carlaviruses, criniviruses, ipomoviruses, and torradoviruses [9,10,11,12,13]. Chemical pesticides are the most widely used method to control *B. tabaci* infestation. Chemical pesticides with a similar mode of action, such as neonicotinoids and insect growth regulators are conventional means to manage *B. tabaci* [14]. The excessive use of these chemicals has led to numerous problems, such as health risk to users and consumers of farm produce, the development of pest resistance, and the destruction of non-target organisms. In recent years, researchers have shown an increasing interest in using biological control agents including entomopathogenic fungi (EPF) as an alternative to chemical control measures [15]. Over the last five decades, biological control measures have been successfully used to control whiteflies in a protected environment [16]. 

In the integrated pest management (IPM) system, EPF have long been recognized as the natural enemies of the insect population [17]. Species of EPF from several genera have been demonstrated to cause natural mortality of the *B. tabaci* population, with more than 20 species identified to be effective against this insect [5,18,19,20]. Species such as *Beauveria bassiana, Metarhizium anisopliae, Isaria fumosoroseus, Ashersonia* spp., and *Verticillium lecanii* are the most common EPF with potentials as biocontrol agents for *B. tabaci* [21,22,23,24]. EPF are known to infect and kill all life stages of *B. tabaci* [25]. This paper is therefore intended to discuss the management of *B. tabaci*, with special reference to biological control using EPF as a component of an IPM system.

## 2. Taxonomy, Origin, and Distribution of Whitefly, *B. tabaci*

*Bemisia tabaci* was first reported and named *Aleyrodes tabaci* by Gennadius in 1889, as a pest of tobacco in Greece. Currently, it is distributed worldwide inhabiting every continent of the world except Antarctica [1,6,26]. In contrast, some European countries, such as Finland, Sweden, the Republic of Ireland, and the United Kingdom, are still yet to report the existence of *B. tabaci* [27].

Whitefly taxonomy is based exclusively on puparial characteristics; however, very little taxonomic information can be found on non-puparial life stages [28]. Sexual dimorphism in these puparia contributes to existing knowledge of the whitefly’s taxonomic tools of identification, development, reproductive potential, and management [29]. The family Aleyrodidae is divided into three subfamilies: Udamoselinae, Aleyrodinae, and Aleurodicinae [30,31]. 

In recent years, important progress has been made at the taxonomic level based on the analysis of the mitochondrial cytochrome oxidase subunit I (mt COI) gene, with at least 43 species complexes of *B. tabaci* identified [26,30,32,33,34]. The Middle East–Asia Minor 1 (MEAM1) and Mediterranean (MED) complexes (previously known as B biotype and *B. argentifolii,* and Q biotype, respectively) are considered the most invasive species with a broad host range of plants [1,32,35]. The nature of their broad host range and the global trade of *B. tabaci* host plants may have contributed to their worldwide distribution [32]. 

## 3. Biology of Whitefly, *B. tabaci*

Whiteflies have a characteristic life cycle of six stages: the egg, four immature stages (nymphal instars), and the adult stage [1]. Temperature, relative humidity, and host plants are the main factors that greatly influence the life cycle of whitefly species [36,37,38]. *Bemisia tabaci* deposit eggs on the upper and lower leaf surfaces of plants, and the number of eggs deposited is significantly affected by temperature, with 28 °C being the most favorable for *B. tabaci* production [36]. Eggs laid are pear-shaped (approximately 0.2 mm long), with a gleaming white color that darkens over time, and usually incubate for about 5–9 days depending on the host species, temperature, and humidity [27,39]. Soon after hatching, the first instar (crawler) travels to a short distance until it successfully probes the leaf to feed on the phloem sap before undergoing three more nymphal instar stages (second, third, and fourth) [40].

During the second instar stage, the whitish-yellow nymphs turn yellowish and dome-shaped after feeding. The pale yellow freshly molted third instar nymphs, however, gradually turn dark yellow and more flattened in shape after feeding [41]. The fourth instar nymphs have a yellowish-white color with large eyes visible through the integument; this stage is also known as the “pupal” stage or “red-eye nymph” [1,39,41].

Fully developed adults of *B. tabaci* emerge from the dorsal surface of the pupal case through an inverted “T”-shaped slit [41]. An adult is yellow-bodied with a pair of white wings that form an inverted V-shape covering the thorax and abdomen. The abdomen of a *B. tabaci* female is large and round-shaped, while that of the male is pointed [1,42]. The entire life cycle of *B. tabaci* from egg to adult takes approximately 16 to 31 days, with some differences between the duration of each stage depending on the host plants used to rear them [38,43]. The Q biotype of *B. tabaci* has been found to have a shorter life cycle and longer adult longevity than the B biotype [44].

## 4. Damage and Losses Caused by *B. tabaci*

*Bemisia tabaci* can cause significant economic losses to crops by causing damage to the host plants during feeding through secretion of honeydew and transmission of plant viruses [45]. Both nymphs and adults of *B. tabaci* cause damage by inserting their mouthparts into the plants during feeding and by transmitting a large number of viruses that can severely damage susceptible plants species [27].

### 4.1. Feeding Damage

It has been found that *B. tabaci* nymphs can inject enzymes that cause changes in plant physiology, leading to irregular ripening of fruit and retarded internal coloration [40]. The honeydew excreted by *B. tabaci* provides a medium for the growth of sooty mold on the leaves and fruits, thus reducing photosynthetic activities, which could negatively affect the quality of farm produce [1,39]. In addition, the feeding of *B. tabaci* on leaves can cause yellowing and crumpling, which subsequently results in stunted plant growth and deformed fruits [3].

### 4.2. Bemisia tabaci as a Virus Vector

More than 200 plant viruses are able to be transmitted by *B. tabaci*, with the majority of these viruses belonging to the genera Begomovirus, Carlavirus, Crinivirus, Ipomovirus, and Torradovirus [46,47,48]. Some of the most vulnerable crops to these viruses are cassava, cotton, cowpea, cucurbits, crucifers, eggplants, tobacco, tomato, potato, soybean, sweet potato, okra, lettuce, pea, bean, pepper, poinsettia, and chrysanthemum [41,46]. Of all the viruses transmitted by *B. tabaci*, begomoviruses are well known as the leading cause of yield losses in crops, ranging from 20–100% and losses worth millions of dollars [27].

Cassava mosaic and cassava brown streak are destructive viral diseases of cassava in Africa spread by *B. tabaci,* affecting approximately half of cassava plants in the country, with annual yield losses of more than 1 billion USD [49]. Several different begomovirus species, such as *Cotton leaf curl Burewala virus* (CLCuBuV), *Cotton leaf curl Multan virus* (ClCuMuV), and *Cotton leaf curl Kokhran virus* (CLCuKoV), cause cotton leaf curl disease complex, which is another example of whitefly-transmitted viruses causing losses amounting to millions of US dollars annually throughout the world, making it the most devastating global disease of cotton [7,50]. In addition to cassava and cotton, global tomato production has also been severely affected by whitefly-transmitted begomoviruses, particularly the species *Tomato yellow leaf curl virus* (TYLCV) [51].

The method of transmission provides knowledge about the periods of virus acquisition and inoculation, which can be used to develop effective management strategies [52]. Plant viruses, such as criniviruses, carlaviruses, ipomoviruses, and torradoviruses, are transmitted in a semi-persistent manner, while the begomoviruses are transmitted in a persistent circulative manner [52,53,54,55]. Semi-persistent transmission of viruses usually requires at least 15 min of acquisition access with a retention time of up to days in the foregut [52,56]. In contrast, several hours are required for acquisition access in the persistent transmission, with retention time in the hemolymph of up to the entire life of the vector [9,55]. In the persistent transmission mode, virus moves from the foregut and into the hemolymph through the midgut of *B. tabaci* before being transported into salivary glands to be egested with saliva into the plant tissues [56].

## 5. Control and Management of Whitefly

IPM is an internationally recognized approach to pest control and is intended to reduce ecological and health damage caused by chemical pesticides. The IPM program for *B. tabaci* includes biological control, crop plant resistance, physical and mechanical methods, and using selective chemical pesticides when necessary [57].

Host plant resistance to whiteflies has been successfully developed due to the growing concern over the increasing use of synthetic chemical pesticides. Cultivars from different varieties of cotton, tomato, and other field crops have been screened against *B. tabaci* and many other sap-sucking insects [58,59,60,61,62]. However, the selection and development of resistant cultivars against whitefly-transmitted viruses in breeding programs are quite challenging, because there is a need to screen and inoculate large numbers of plants to select genotypes with resistance genes [9].

Physical and mechanical methods are techniques emphasizing the creation of unfavorable environments for pests, which include the removal of pest breeding sites and the use of healthy seedlings and resistant varieties [63]. Cultural methods such as crop rotation could increase host periods or reduce intercrop migrations through careful consideration of the types and special arrangement of planted crops, thus, ultimately leading to the control of the *B. tabaci* population [11]. The application of an electric field screen to greenhouse windows can prevent the entry of whitefly, but requires the presence of a guard at the greenhouse entrance area [64,65].

Based on the principle of IPM, pesticides should be the last choice for farmers to use when other options are not successful against the infestation of pests in crops [66]. Over the past two decades, insecticides, including nicotinoids and insect growth regulators, have demonstrated physical and immediate action in controlling *B. tabaci* and other pest-sucking insects [67,68]. Foliar applications of systemic insecticides in the neonicotinoid class such as clothianidin, dinotefuran, imidacloprid, thiamethoxam, chlorantraniliprole, spinosad, and flupyraifurone can provide sufficient control of whitefly [14,69,70]. The effect of chemical pesticides on the non-target organism, environmental contamination, and resistance of insect pests have led to research on biological control agents as alternative control measures. *B. tabaci* can be effectively controlled by integrating multiple biological control agents such as parasitoids, predators, and EPF [45,71,72,73,74,75,76].

There are at least 115 species of whitefly parasitoids belonging to 23 genera in five families: Aphelinidae, Azotidae, Encyrtidae, Signiphoridae (Chalcidoidea), and Platygastridae (Platygastroidea) [77]. Two genera, *Encarsia* and *Eretmocerus* in the order Hymenoptera, are the most well-known whitefly parasitoids found throughout the world, while others are specific to different continents [78]. These two parasitoids have been reported to significantly lower the population of *B. tabaci* via parasitism and host feeding [79,80,81]. Moreover, there are approximately 150 arthropod species currently described as predators of whiteflies, and the majority of them are ladybird beetles, predaceous bugs, lacewings, phytoseiid mites, and spiders [79]. The biological control study of predators reported by Nomikou et al. [82] showed that two phytoseiid species, *Euseius scutalis* (Athias-Henriot) and *Typhlodromips swirskii* (Athias-Henriot), can significantly suppress *B. tabaci* population on a single plant.

## 6. Biological Control of *B. tabaci* with Entomopathogenic Fungi

EPF, an important group of biological control agents for whiteflies, other sap-sucking pests, and pests with chewing mouthparts, play a key role in the natural mortality of whitefly populations, as they can directly infect insects through the cuticle [5,83]. There are approximately 700 species of EPF belonging to the group Laboulbeniales and Pyrenomycetes (phylum Ascomycota), Hyphomycetes (phylum Deuteromycota), and Zygomycetes (phylum Zygomycota) [84]. Most of the EPF currently being studied belong to the class Entomophthorales of the phylum Zygomycota and Hyphomycetes of the phylum Deuteromycota [76].

Based on previous research findings, EPF can be isolated from insect cadavers or soil and can be grown in artificial media [19,85,86]. Solid-state, liquid-state, and di-phasic fermentation can be used for large-scale production of EPF by using conidiophores or conidia and hyphae on a granular substrate [87]. The spray and dip application is the most common method used in controlling *B. tabaci*, with many promising results (Table 1). Various bioassay methods have been developed to evaluate the efficacy of EPF, and the majority of them were applied in the form of either spraying or dipping on *B. tabaci* [20].

Common species of EPF (*B. bassiana, M. anisopliae,* and *I. fumosorosea*) with potential importance in biological control have been commercially produced and documented [88,89]. EPF that infect and kill all developmental stages of the whitefly species complex are mostly derived from the genera *Verticillium, Isaria,* and *Aschersonia* [25]. Most of the EPF involved in *B. tabaci* control studies were species from the following genera: *Aschersonia, Beauveria, Isaria* (*Paecilomyces*), *Lecanicillium* (formerly *Verticillium*), and *Metarhizium* [1]. Moreover, it has been recently reported that *Clonostachys rosea* has a pathogenic effect on the fourth instar nymphal and adult stages of *B. tabaci* [90].

Biological control of *B. tabaci* using EPF is summarized in Table 1. Overall findings from previous studies indicated that all species of EPF were pathogenic to *B*. *tabaci*, with the second instar stage being the most susceptible to EPF infection. Species from the genus *Aschersonia* have long been used for controlling whiteflies and other related greenhouse insects in the environment, with high relative humidity and moderate temperatures [5,91]. The application of *Aschersonia aleyrodis* to control *B. tabaci* is a promising EPF candidate, which has been proved effective in parasitizing whiteflies [92]. The effectiveness of *A. aleyrodis* isolates tested against second, third, and fourth instar of MEAM1 *B. tabaci* under laboratory and greenhouse conditions showed greater than 50% *B. tabaci* mortality for seven days [24]. The survival rate of the first, second, and third instar nymphs of *B. tabaci* can be significantly reduced through the application of *A. aleyrodis* isolate (Aa005) under glasshouse conditions [93]. A pathogenic strain of *A. aleyrodis* (Aa-J18) could kill nymphs and adults of *B. tabaci* with a mortality of up to 99%, as reported by Prayogo and Bayu [94].

*Beauveria bassiana* (Balsamo-Crivelli) Vuillemin is one of the most commonly encountered EPF, and has been commercially developed as a microbial insecticide to control *B. tabaci* [1,95]. Zafar et al. [96] applied three different isolates (Bb-01, Bb-08, and Bb-10) of *B. bassiana* against eggs and the second nymphal instar of *B. tabaci* on four host plants (*Gossypium hirsutum, Lycopersicum esculentum, Solanum melongena,* and *Capsicum annum*). The results showed a significant reduction of *B. tabaci* eggs and nymphs. Meanwhile, Prithiva et al. [97] demonstrated the effectiveness of three formulations (oil formulation, talc formulation, and crude formulation) of *B. bassiana* isolate against *B. tabaci* on tomato under microplot conditions. The study showed a reduction in population over control, with the oil formulation being the most effective against *B. tabaci*. The interaction of *B. bassiana* with other biological control agents, such as *Bacillus thuringiensis* for the biological control of *B. tabaci*, were shown to have an antagonistic effect, and mortality greater than 50% was observed over a period of 7 days [98]. A combination of bacterial biosurfactant with two EPF, *Cordyceps javanica* and *B. bassiana*, has recently been found to cause 100% mortality of the third instar nymphs of *B. tabaci* within 4 days [99]. Likewise, the combination of *B. bassiana* and some plant extracts such as neem have shown increased mortality of *B. tabaci* [100,101,102,103].

*Isaria fumosoroseus* (*Paecilomyces fumosoroseus*) is one of the most important natural enemies of whiteflies, and it can cause an epizootic in *B. tabaci* in greenhouse and open field environments [104]. Currently, *I. fumosoroseus* is globally distributed and can infect a broad range of pests in agricultural and forest areas [105]. Commercial demand to investigate bioproducts based on *I. fumosoroseus*, due to its capacity to cause natural epizootics on several insect pests, is therefore compelling [106]. Although many studies on the potential of *I. fumosoroseus* against *B. tabaci* have been carried out [6,21,107,108], they have been largely focused on the mortality and infections of nymphs and adults of *B. tabaci* (Table 1) [104]. The efficacy of *I. fumosoroseus* in immediate control of the *B. tabaci* population can be significantly improved by adding synergistic chemicals, such as imidacloprid and thiamethoxam [109].

*Lecanicillium lecanii*, also known as *L. muscarium* (formerly known as *V. lecanii*), is one of the most important commercialized EPF, and has long been used to control greenhouse insect pests, including *B. tabaci* [110]. It has been proved to be pathogenic to all developmental stages of *B. tabaci,* with the most susceptible stage of infection occurring at the second instar nymph stage [1,110]. An experiment was conducted to determine the pathogenicity of *L. lecanii* and other EPF *B. bassiana*, *M. anisopliae,* and *M. rileyi* against *B. tabaci*. Isolates of *L. lecanii* were found to be the most virulent among all the fungal isolates tested [23,111]. Compatibility experiments of *L*. *lecanii* and other chemical insecticides such as imidacloprid, buprofezin, and teflubenzuron showed promising outcomes in reducing the population of different stages of *B. tabaci* [112,113,114].

*Metarhizium anisopliae* was first discovered to be effective against the greenhouse whitefly, *Trioleurodes vaporariorum* [115]. However, it has recently been shown that *M. anisopliae* can potentially infect all developmental stages of different whitefly species [116,117]. The efficacy of five *M. anisopliae* strains (PR1, GT2, TFFH3, GJ4, HSAH5) against *B. tabaci* infesting *S. melongena* showed lethal infections of *B. tabaci* in a dosage response assay, whereby the infectivity rate for the second nymphal instar was 12 times higher than the fourth instar upon inoculation with strain GJ4 [118]. The pathogenicity of six isolates of *M. anisopliae* was tested on the *B. tabaci* Q biotype, where the results showed greater than 50% mortality in all the six isolates [119]. However, higher mortality of *B. tabaci* (97%) was observed under osmotic conditions upon infection with *M. anisopliae* isolated from *Coptotermes gestroi* (Rhinotermitidae: Isoptera) [120].

## 7. The Infection Process and Life Cycle of Entomopathogenic Fungi on *B. tabaci*

**Figure 1:**
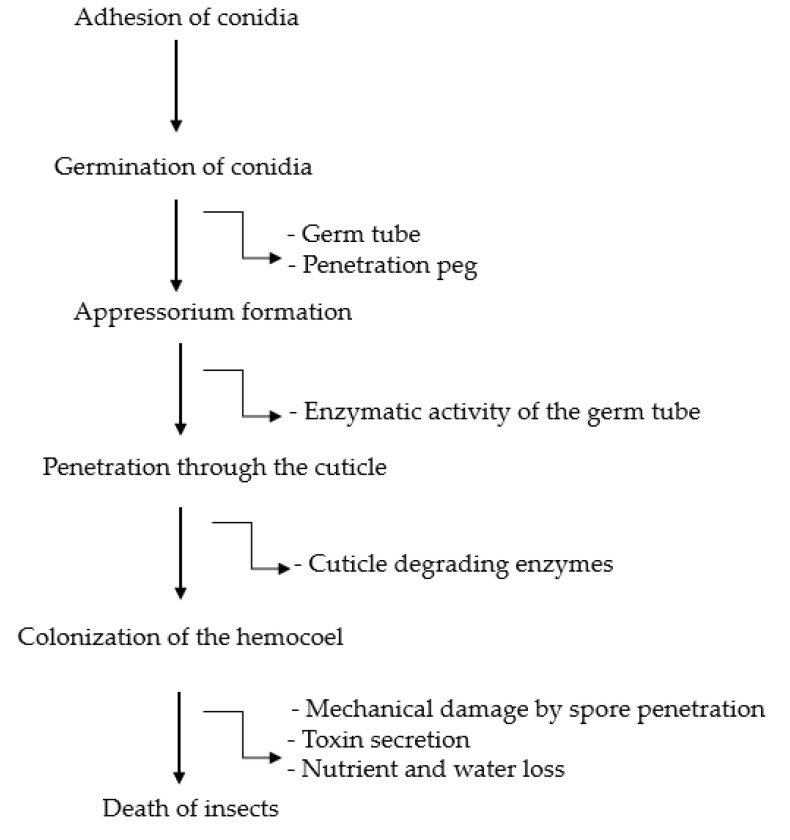
Diagrammatic representation of the EPF infection process on insects.

The insect cuticle is an important structure in the infection process of EPF as it is the main route for fungus penetration [89]. The steps involved in the infection process of EPF on *B. tabaci* are summarized in Figure 1. The fungus must first adhere to and interact with the epicuticular layer of the host by developing physical or enzymatic activities upon penetration into the insect cuticle [89,130]. However, some insects have a substance that can inhibit or promote conidia attachment or germination [84,130]. Attachment and germination of fungal spores start once they have landed on the insect cuticle. The pathogenic interaction of the EPF and the insect is established by the formation of an infective structure called the appressorium [131], which penetrates into the insect cuticle using mechanical pressure and cuticle-degrading enzymes [132]. The mechanical damage resulting from EPF penetration, toxicosis (toxins produced by the EPF), and nutrient exhaustion, leads to death of the insect [75,133]. The EPF grow sporadically within the insect hemocoel, and hyphae colonization of the cadaver following the death of insect.

The life cycle of EPF synchronizes with the insect life stages and environmental conditions [75,76]. Generally, the life cycle consists of a parasitic phase (from host infection to its death) and the saprophytic phase (after host death) [134]. The life cycle begins with the germination of spores following adhesion to the insect body [133]. The penetration of EPF into the insect body is a result of mechanical and physiological enzymatic activity of the germ tube [133]. Once the spores penetrate the insect body, yeast-like propagules are produced by budding-like growth and are distributed throughout the hemocoel [83]. Besides producing toxins, EPF can disrupt the metabolic processes of the insects through spores invading organs, thus resulting in the insect death [132]. Upon the insect death, the fungus colonizes the cadaver and reverts into the typical hyphal form (the saprophytic phase) due to sporulation [83,134]. The spores are then spread passively from the fungus-infected cadaver to new hosts [133].

In general, all EPF have the same mode of infection, including attachment of spores to the cuticle, germination of hyphae over the surface of insects, penetration of hyphae through the integument, growth of fungus in the hemocoel, and ultimately death of the whitefly (Figure 1) [89,135].

## 8. Merits and Demerits of Using EPF as Biological Control Measures

The excessive use of chemical pesticides in agriculture has led to environmental contamination, as well as harmful effects on non-target organisms, including beneficial insects (pollinators), livestock, and humans. The application of EPF may, therefore, provide an alternative to the conventional use of chemical pesticides to effectively control insect pests, while preserving the natural environment, which is one of the main goals of sustainable agriculture [75].

The regulation of insect pests and arachnid populations has been significantly enhanced by the use of EPF, due to their major benefits over the use of chemical pesticides, including broad host range, the absence of harmful side effects for non-target organisms, easy mass production, low cost, and eco-friendliness [136,137,138]. Besides being efficient pest and disease control agents, EPF can also serve as plant growth promoters [132,137]. The unique infection mechanism of EPF ensures no issue of insect resistance, so they can be sustainably used as pest control [83]. Butt et al. [139] reported that compounds isolated from fungal biocontrol agents have led to the development of pharmaceutical drugs and safer agrochemicals.

Although EPF offer several advantages over chemical pesticides, there are some limitations to the application of EPF in controlling insect pests. One of the major limitations of EPF is the lengthy duration of 2–3 weeks to kill the insect pest population, whereas chemical pesticides may take only 2–3 h [83]. Environmental factors such as sunlight, temperature, humidity, and UV exposure can affect the insecticidal activity of EPF against insect pests in open fields of tropical regions [107]. The effectiveness of EPF in field trials with an uncontrolled environment may be hindered, although they can be successfully used in a controlled environment like a glasshouse [75]. Additionally, research related to the development of biocontrol agents or natural products has gained less popularity in terms of investment, in comparison to that concerning chemical pesticides [139].

## 9. Conclusions

*Bemisia tabaci* is considered a destructive insect pest of numerous crops around the world. Crops are affected directly by *B. tabaci* during feeding, and indirectly through the transmission of viral diseases, which can cause severe crop damage and yield losses worth millions of dollars. The control of *B. tabaci* relies heavily on pesticides despite several drawbacks, such as insecticide resistance and health risk to farmers and consumers. Hence, the IPM approach is deemed a safer and effective control measure to control *B. tabaci*, and includes the use of biological control, based exclusively on the effective use of natural parasites (parasitoids), predators, and entomopathogens.

The application of EPF as an effective biological control method of *B. tabaci* has been well demonstrated in various studies. The most common and popular EPF used to control *B. tabaci* are *Ashersonia* spp., *B. bassiana, I. fumosoroseus, M. anisopliae,* and *Verticillium lecanii*, each subject to various reviews. High populations of EPF and their effectiveness against *B. tabaci* can be sustained by improving conidia formulation and substrate. These efforts may increase the stability of EPF propagules and reduce the time required to kill *B. tabaci.* Moreover, some EPF, such as *B. bassiana* and *M. anisopliae* are associated with plants as symbiotic endophytes, which may help in the development of more effective insect pest management strategies. Endophytic inoculation of EPF in different parts of plants (foliar, root, seed, and stems) to effectively control *B. tabaci* could be utilized for the development of new IPM strategies. Taken together, EPF have a promising future in the sustainable control of *B. tabaci*.

## Figures and Tables

**Table 1:** Summary of control methods of *B. tabaci* using entomopathogenic fungi (EPF).

Species	Bioassay Method	Significant Effects/Results	Country Where Work Was Conducted	Ref.
*Aschersonia aleyrodis*	Second and third instars on eggplant leaves were sprayed with fungal spores at 1 × 10^7^ conidia/mL.	Mortality: Second instar: 71.21% Third instar: 69.31% Pupae: 53.36% LC_50_: Second instar: 7.93 × 10^6^; third instar: 1.08 × 10^7^; pupae: 1.56 × 10^7^ conidia/mL LT_50_: 4.60 days for second instar.	China	[24]
*A. aleyrodis*	First, second, and third instars on eggplant were sprayed with spores at 1 × 10^7^ conidia/mL on eggplant leaves.	The survival of first, second, and third nymphal instars was significantly affected.	China	[93]
*A. aleyrodis*	Eggs; first, second, third, fourth instars; and adults on the leaves of soybean were sprayed with 1 × 10^6^ conidia/mL.	The highest mortality (99%) was observed for the first, second, and third instars and the lowest mortality in the adult stage. LD_50_: 6–7 × 10^6^ conidia/mL LT_50_: 3.50–3.75 days for nymph stage; 4.50 days for adults.	Indonesia	[94]
*A. placenta*	First, second, and third instars on tomato were sprayed with 1.5 mL of fungal suspension.	Mortality ranged from 93% to 100%. LD_50_ and LD_90_ values decreased with time and increased with instar. LT_50_ values decreased with conidial concentrations.	China	[121]
*Beauveria bassiana*	Eggs and nymphs were sprayed with different concentrations on plant leaves of cotton, tomato, eggplant, and bell pepper.	Most effective isolate (Bb-01) on cotton mortality: eggs: 65.30% nymphs: 88.82% LC_50_ value: 2.4 × 10^7^ spores/mL. LT_50_: lowest on cotton, 5.40 days	Pakistan	[96]
*B. bassiana*	Nymphs and adults on tomato leaves were sprayed with different formulations of 10^8^ spores/mL.	Reduction of the population over control in formulations: Oil: 45.86%. Talc: 29.62%. Crude: 21.63%.	India	[97]
*B. bassiana*	Eggs and first, second, third, and fourth instars were immersed in 1 mL of conidia suspension for 10 min.	First and second instars were more susceptible than the third and fourth instars. Nymphs were highly susceptible compared to eggs.	Saudi Arabia	[95]
*B. bassiana*	Fourth instars from cucumber, tomato, melon, green pepper, potato, eggplant, marrow, cabbage, bean, and cotton plants were immersed in 1.0 × 10^7^ conidia/mL for 10 s.	Mortality and average survival time after 8 days of inoculation were significantly influenced by the host plants. Mean mortality ranged between 52.3 ± 7.3 for nymphs reared on cotton and 91.8 ± 5.8 for nymphs reared on cucumber.	Spain	[122]
*B. bassiana*	Adult *B. tabaci* (2–3 days old) on cotton were sprayed with three concentrations (1 × 10^3^, 1 × 10^5^, and 1 × 10^7^ spores/mL) of 1 mL of fungal suspension.	Mortality recorded at the lowest dose (1 × 10^3^ spores/mL) was 11%, while the highest percentage mortality (56%) was recorded at a high dose of 1 × 10^7^ spores/mL, and the recorded natural mortality was only 5%.	Egypt	[123]
*Isaria* spp.	Second, third, and fourth instars were sprayed with spore concentrations in clip-screen cages on sweet potatoes.	LC_50_ and LT_50_ values when exposed to 1000 spores/mm^2^: LC_50_: second instar: 72–118 spores/mm^2^; third instar: 166–295 spores/mm^2^; fourth instar: 166–295 spores/mm^2^ LT_50_: second instar: 3 days; third instar: 4 days.	USA	[124]
*I. fumosoroseus*	Eggs and first, second, third, and fourth instars on eggplants were dipped in conidia suspension (1 × 10^6^ conidia/mL) for 2–3 s.	Most effective isolate (UPM isolate) mortality: Eggs: 91% Second instar: 90% Third instar: 86% Fourth instar: 89% LT_50_: 3.94 to 6.28 days.	Malaysia	[108]
*I. fumosoroseus*	First, second, and third instars on cucumbers were sprayed with spores at 1 × 10^7^ conidia/mL.	The second instar was the most susceptible life stage with mortality rate at 83% after 7 days of application.	China	[125]
*I. fumosoroseus*	Second instars on eggplants were dipped in five different concentrations (1 × 10^3^,1 × 10^4^,1 × 10^5^,1 × 10^6^,1 × 10^7^ conidia/mL) for 20 s.	LC_50_ values: 1.10 × 10^4^ conidia/mL after 12 days of treatment. At a concentration of 1 × 10^7^ conidia/mL, minimum average longevity and number of progenies produced were 9 days and 10.92 eggs/female, respectively, as compared to 16.3 days and 83.67 eggs/female for the control.	China	[104]
*Lecanicillium lecanii*	Second instars on five host plants were sprayed with 10^7^ conidia/mL.	The highest mortality was recorded in all host plants: Laboratory conditions: nymphal mortality: >90% Glasshouse conditions: nymphal mortality: 81%.	United Kingdom	[110]
*L. lecanii*	Toxin emulsion was applied to female adult *B. tabaci* on the tomato plants.	The toxin reduced the hatching of whitefly eggs, the survival rate of the nymphs, and the emergence and fecundity of the progeny adults.	China	[126]
*L. muscarium*	First, second, third, and fourth instars on tomato and verbena plants were sprayed with a fungal suspension (10^7^ spores/mL).	First and second instar nymphs were more susceptible to *L. muscarium* than the third and fourth instar nymphs.	United Kingdom	[127]
*Metarhizium anisopliae*	Second instar nymphs were sprayed with 10^7^ spores/mL of three vegetable oil formulations.	The highest mortality was observed with sunflower oil followed by olive oil and maize oil formulations.	Argentina	[2]
*M. anisopliae*	Second instar nymphs on eggplants were dipped into 10^8^ conidia/mL for 10 s.	Mortality caused by two isolates under osmotic conditions was 83.9% and 83.8%.	Malaysia	[120]
*M. anisopliae*	Second and fourth instars on brinjals were sprayed with 2 mL of 10^7^, 10^5^, 10^3^, and 10 conidia/mL.	LC_50_ value: Lowest on second instar, 6.62 × 10 conidia/mL. LT_50_: 2.25 days	Malaysia	[118]
*M. anisopliae*	Second instars on eggplants were dipped into 10^8^ conidia/mL for 10 s.	The highest mortality of 84.3% was observed in the isolate GT3.	Malaysia	[119]
*B. bassiana, I. fumosorosea,* and *L. muscarium*	Nymphs were sprayed with conidia at 10^7^ conidia/mL (150 conidia/mm^2^).	All fungi isolates were pathogenic to whitefly nymphs. Isolates of *B. bassiana* and *I. fumosorosea* were significantly more virulent than that of *L. muscarium,* with > 77% nymphal mortalities.	Brazil	[5]
*B. bassiana, M. anisopliae*, and *I. fumosorosea*	Conidia at 5 × 10^9^ conidia/ha were sprayed on eggs; first, second, third, and fourth instars, and adult *B. tabaci* on the underside of the leaves to the point of runoff.	There were no significant differences in individual mortality for all life stages between the different strata (the top, middle, and bottom thirds). *M. anisopliae* was significantly more effective against eggs; first, second, and third instar nymphs; and pupae.	Mexico	[117]
*B. bassiana* and *L. lecanii*	Nymphs on tomato plants were sprayed with fungal filtrate, conidia, and filtrate + conidia of two fungal strains.	In all the three bioassays, the isolate BB-72 was the most virulent, causing high mortality using all three different concentrations of the two fungal strains tested.	China	[128]
*M. anisopliae*, *B. bassiana*, and *V. lecanii*	Three different concentrations (1 × 10^7^, 1 × 10^8^, and 1 × 10^9^ spores/mL) were sprayed onto adult *B*. *tabaci.*	The percentage of reduction ranged between 52% and 100% in all concentrations. All the treatments caused 100% mortality with concentrations of 1 × 10^9^ after the sixth day of application.	Egypt	[23]
*M. anisopliae, B. bassiana,* and *V. lecanii*	Three different concentrations (2 × 10^3^, 2 × 10^4^, and 2 × 10^5^ conidia/mL) were sprayed onto adult *B*. *tabaci.*	The percentage of mortality ranged between 80–100% (*V. lecanii)*, 45–75% (*B. bassiana)*, and 45–70% (*M. anisopliae)* on the seventh day after treatment. The concentration of 2 × 10^5^ conidia/mL was highly toxic under both laboratory and field conditions.	Egypt	[129]

Ref = reference; LC_50_ = lethal concentration required to kill 50%; LT_50_ = lethal time required to kill 50%.
